# Reducing the Application Rate of Molluscicide Pellets for the Invasive Spanish Slug, *Arion vulgaris*

**DOI:** 10.3390/insects13030301

**Published:** 2022-03-17

**Authors:** Mantas Adomaitis, Grita Skujienė, Paulius Račinskas

**Affiliations:** Department of Zoology, Institute of Biosciences, Life Sciences Centre, Vilnius University, Saulėtekio Av. 7, LT-10257 Vilnius, Lithuania; grita.skujiene@gf.vu.lt (G.S.); racinskas.p@gmail.com (P.R.)

**Keywords:** *Arion vulgaris*, metaldehyde, iron phosphate, mollusk, control

## Abstract

**Simple Summary:**

*Arion vulgaris* has become a major invasive pest slug in Europe, causing extensive damage to many crops. To control this pest, the use of chemical molluscicides remains the most important. However, despite the proved efficacy, they still have detrimental environmental effects. We performed two double-replicated laboratory studies testing molluscicide pellets with metaldehyde (3% and 5%) and iron phosphate (1%) and found the reluctance of slugs to eat a full lethal dose regardless of whether the poison is stronger or weaker. As a consequence, slugs remain alive and only reduce their herbivory by half; the remaining granules or their parts are the main source of toxic effects of molluscicides in the environment. Moreover, a higher metaldehyde content of the pellets does not lead to lower herbivory. The results showed that a new application of molluscicides could be useful; the application rate should be decreased according to the ability of slugs to eat a certain amount of molluscicide pellets.

**Abstract:**

*Arion vulgaris* are mostly controlled using chemical molluscicide products, and the detrimental environmental effects of these molluscicides can be reduced by decreasing the number of pellets applied per unit area. The objective of this study was to compare three slug control methods during two double-replicated seven-day laboratory experiments, in which slugs could choose the number of pellets with metaldehyde (3% or 5%) or iron phosphate (1%) and different types of food: leafy plants (lettuce), root vegetables (carrot), a cereal-based diet (oatmeal), or an animal-based diet (dry cat food). Slugs were irrigated and allowed to recover. We found a reluctance of slugs to eat big amounts of pellets and, therefore, to reach a full lethal dose, which resulted in low mortality (the rate was only 2.1%), regardless of whether the poison was stronger or weaker. Herbivory of slugs was in some cases reduced by half, but no treatments resulted in slugs to stop eating. Pellets with 3% metaldehyde were significantly more acceptable than pellets with 5% metaldehyde (uneaten pellets were left). The results showed that the new application of molluscicides could be useful; the application rate should be decreased according to the slugs number and ability of slugs to eat a certain amount of molluscicide pellets.

## 1. Introduction

During the last two decades, the invasive Spanish slug *Arion vulgaris* Moquin-Tandon 1855 (formerly known as *Arion lusitanicus* auct. Non-Mabille) (Gastropoda: Stylommatophora: Arionidae) has become the most harmful pest for private home gardens and ecological agriculture in Europe [1,2]. The findings in the anthropogenic landscape undoubtedly justify the spread of Spanish slugs through global trade routes when warm and humid winters and frequent rains during plant growth periods influence successful breeding conditions and survival [3,4]. A slightly warmer climate, with reduced seasonal amplitude, increased precipitation, and reduced ice cover, has been observed during the past five to seven decades [5]. Spanish slugs feed on many vegetables, wheat, fruits, and ornamental plants [6,7,8] and leave gnawed plant remains and slime. Control methods in Europe include chemical biocides that comply with Regulation (EU) 528/2012 of the European Parliament and of the Council. Only two chemicals have been permitted for use in Europe for slug and snail bait and are applied as pellets for use in home gardens and on food and seed crops: metaldehyde and iron phosphate. Some negative effects of these molluscicides on non-target organisms have been reported [9,10]. Metaldehyde presents a large water pollution risk due to its mobility in soil once leached from slug pellets on agricultural land [11]. Therefore, the excessive application of molluscicides should be avoided. There are quite efficient non-chemical and biocontrol methods widely used in EU, such as physical barriers (diatomaceous earth, hydrated lime, sulfur, fumed silica, wood ash) [12,13], agronomic practices, and biological (nematodes) and bio-rational control [14], but it is often simpler to apply chemical methods.

The effectiveness of the ingredients of molluscicides has been studied in several countries, and various causes have been identified to reduce their effectiveness. Some of the reasons were related to applying the bait at the right time and at sufficient pellet density [15,16,17,18]. For example, the most effective application was in the spring after juvenile slugs become active [19], but metaldehyde pellets were not effective when the density exceeded 33 slugs per square meter [18]. Second, wet conditions could accelerate the degradation of the molluscicide pellets and increase the recovery of slugs from poisoning [15,16,17,18,20,21]. Third, the slug age has been shown to be crucial: juveniles and subadults were less susceptible to poisoning than adults [22].

It was determined that larger molluscicide pellets are less attractive and less effective [18]. One of the most unexpected reasons for the reduced effectiveness of the poison was earthworms. Field data have shown that an average of 17% of all bait pellet types were removed nightly by earthworms; after 5.1–6.4 days, 100% of the pellets disappeared, regardless of the chemical composition of the bait pellet [23]. However, such earthworm viability was not confirmed under other food attractiveness [20].

Several studies have tested the efficacy of molluscicides on slug mortality and herbivory, but none have investigated the efficacy on *A. vulgaris* with multiple food choices in relatively humid conditions [17,24,25]. The objective of this study was to compare three slug control methods during a double-replicated seven-day laboratory experiment when slugs can choose the number of pellets and different types of food that they consume: leafy plants (lettuce), root vegetables (carrot), a cereal-based diet (oatmeal), or an animal-based diet (dry cat food). We hypothesized that the opportunity for slugs to freely choose the number of pellets (from one to five per session) and food type will reduce the effects of metaldehyde or iron phosphate pellets.

## 2. Materials and Methods

### 2.1. Collection and Maintenance of Slugs

On four dates—August 6, 13, 20 and 26, 2018—80 adult *A. vulgaris* of a similar size (4.5 ± 0.69 g) were hand-collected in the Vilnius University Botanical Garden (54°44′9.88″ N, 25°24′19.88″ E). The collected slugs were kept in plastic jars and pre-starved for 24 h before the trial; they were then introduced into the experiment. The pre-starvation and the trial were performed in the Zoology Laboratory of the Life Sciences Centre of Vilnius University, where the slugs were maintained in a Fitotron SGC 120 thermo-climate chamber (Weiss Gallenkamp, Loughborough, UK) at 15 °C, with a 12 h light/12 h dark photoperiod and 80% relative humidity. During the trials, the slugs were housed individually in closed transparent plastic containers (100 mm × 100 mm × 45 mm) covered with wet geotextile moistened by water. In order to create conditions that are close to humid and rainy Mid-European weather, the slugs were also irrigated. Ventilation in the containers was provided by making holes in the lids. Slugs could freely move in the containers and eat lettuce, carrots, dry cat pellets, or oatmeal.

### 2.2. Laboratory Trials

There was a total of four experiments (two designs) each conducted over a 7-day period: I (August 7 to 13), II (August 14 to 20), III (August 21 to 27), and IV (August 28 to September 3). Each treatment had 20 slugs (one treatment was control, the other three had molluscicide baits); in total, we had 80 experimental units (slugs) per experiment. A total of 320 slugs were used. We used three molluscicide baits approved in Lithuania (Table 1): Ferramol with 1% iron phosphate (W. Neudorff GmbH KG, AS2-58M/2016, Germany), Gusto with 3% metaldehyde (Adama Registrations B.V. AS2-17M/2016, The Netherlands), and Lima Oro with 5% metaldehyde (Sharda Cropchem España AS2-13M (2018), Spain).

Each slug was provided with a full portion of food that comprised 5 g of lettuce (“Iceberg”), 2 g carrot (“Nanto”), one dry cat food pellet (0.2 g) (“Purrrfect”, Czech Republic), and two oatmeal grains (“Skanėja”, Lithuania) per container. Molluscicide pellets were added to the containers in two designs (different doses): 1 pellet (experiments I and II) or 5 pellets (experiments III and IV) three times in each experiment in combination with evaluation of food consumption and cleaning the container and adding a new portion of food (Table 2). Control containers were not subjected to molluscicides.

The herbivory of slugs, pellet ingestion, and visual appearance after pellet ingestion were assessed every second day. After each assessment, uneaten food and molluscicide pellets were removed, the container was replaced with a clean one, and the irrigated slugs were given a new portion of the same food and molluscicide pellet or pellets (Table 2). For a more detailed timetable on the activities during the experiment, see Table 2.

Slugs were weighed with a KERN EMB 600-2 precision balance (KERN and Sohn GmbH, Balingen Germany).

Molluscicide pellet and food consumption (separately for each food item) was calculated as the percentage of the eaten part. Lettuce and carrot consumption was later extrapolated to grams.

Damage to slugs was estimated by a five-point scale: 1—no visible damage; 2—body has some unusual mucus; 3—body has excessive yellow slime secretions; 4—body has excessive orange slime secretions and some body deformation (raised tail, genital protrusion, or other); 5—dead.

### 2.3. Statistical Analysis

Specimens that did not eat molluscicide pellets or that laid eggs during the treatment were excluded from the analysis of the molluscicide effect. Statistical analysis was performed using R (version 3.5.3). All data were tested for normality using the Shapiro-Wilk test. When normality was confirmed, a Levene’s test for equal variances was performed before the estimation of differences between groups using a one-way analysis of variance (ANOVA) and Tukey’s honest significant difference (HSD) post hoc tests. All data with a non-normal distribution (data on food and molluscicide consumption) were log transformed and subsequently checked for equal variances with a Levene’s test before conducting the three-way ANOVA and Tukey’s HSD post hoc tests. Kendall’s tau-b correlation coefficient was used to examine the relationship between food consumption, slug weight, damage to slug health, and portion of bait eaten. All statistical analyses were performed at the significance level of α = 0.05. Graphics were made using Microsoft Excel.

## 3. Results

Slug herbivory and slug weight were significantly influenced by the slug control method (type of molluscicide), design (1 or 5 pellets), and the time of the experiments (such as the first half of August (I and II experiments) and the second half of August (III and IV experiments)) (Table 3). Notably, feeding decreased but did not stop (Table 4), and only five slugs died (a 2.1% mortality rate) during the experiments.

Given the choice to eat different numbers of molluscicide pellets, no slug ate the full dose for use per square meter (Table 1). The majority consumed a dose that was approximately 2–4-times lower than the recommended dose.

**Figure 1:**
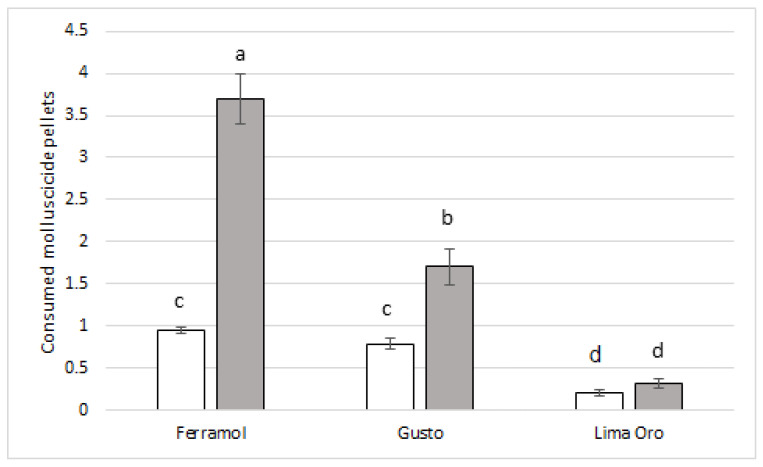
Mean (± standard error of the mean) consumption of molluscicide pellets during the first day of exposure. White bars indicate treatments with 1 pellet, while gray bars indicate treatments with 5 pellets. Ferramol 1 pellet (*n* = 38), Ferramol 5 pellets (*n* = 33), Gusto 1 pellet (*n* = 40), Gusto 5 pellets (*n* = 39), Lima Oro 1 pellet (*n* = 34), and Lima Oro 5 pellets (*n* = 30). Means with different letters are significantly different (Tukey’s HSD, *p* < 0.05).

The molluscicides had unequal acceptability (Figure 1). During the first contact with molluscicides, 100% of slugs consumed one or more Ferramol or Gusto pellet, while only 10% of slugs tasted a Lima Oro pellet (Figure 1). The slugs provided with one molluscicide pellet ate significantly less Lima Oro than Ferramol (*t* = 10.38, *p* < 0.0001) and Gusto (*t* = 8.27, *p* < 0.0001), but they ate similar amounts of Ferramol and Gusto (*t* = 2.29, *p* = 0.06). For the slugs provided with five pellets, the most consumed molluscicide was Ferramol (*t* = 6.49, *p* < 0.0001 compared to Gusto; *t* = 10.27, *p* = 0.0001 compared to Lima Oro), while the least consumed was Lima Oro (*t* = 4.36, *p* < 0.0001 compared to Gusto). Slugs consumed similar amounts of Lima Oro pellets for both designs (one or five pellets; *t* = 1.79, *p* = 0.08) (Figure 1).

All molluscicides significantly decreased slug weight compared to the control group (*p* < 0.001 for all) in both experimental designs, except for slugs given one Ferramol pellet (*t* = 2.53, *p* = 0.059). Among the slugs given one molluscicide pellet, both metaldehyde preparations (Gusto 1 and Lima Oro 1) were more effective than iron phosphate (Ferramol 1) and significantly decreased (*p* < 0.001 for both) slug weights. These differences were not apparent for slugs given five pellets, although there was a moderate negative correlation between the number of ingested molluscicide pellets (1 pellet design) and body mass of slugs only for Lima Oro (Kendall tau-b correlation coefficient = 0.49; *p* < 0.001) (Table 4).

**Figure 2:**
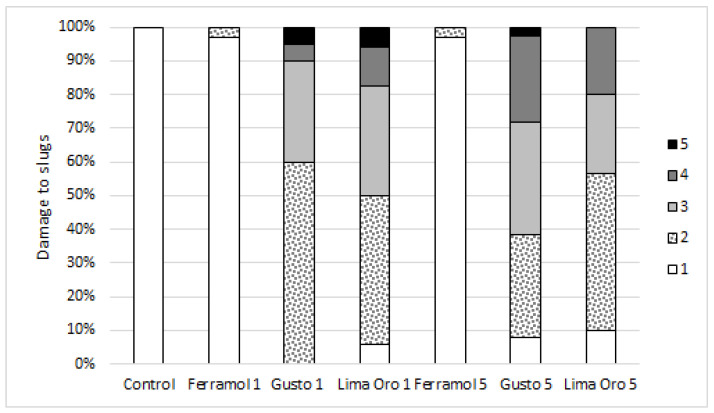
Percentage of damage to slugs (*n* = 320) by molluscicides, expressed in each damage category: 1—no visible damage; 2—body has some mucus; 3—body has excessive yellow slime secretions; 4—body has excessive orange slime secretions and some body deformation (raised tail, genital protrusion, or other); 5—dead. The number next to the molluscicide name on the x-axis indicates the number of pellets (1 or 5) for each treatment.

Only metaldehyde-based molluscicides had an effect on the visible damage of slugs (Figure 2).

**Figure 3:**
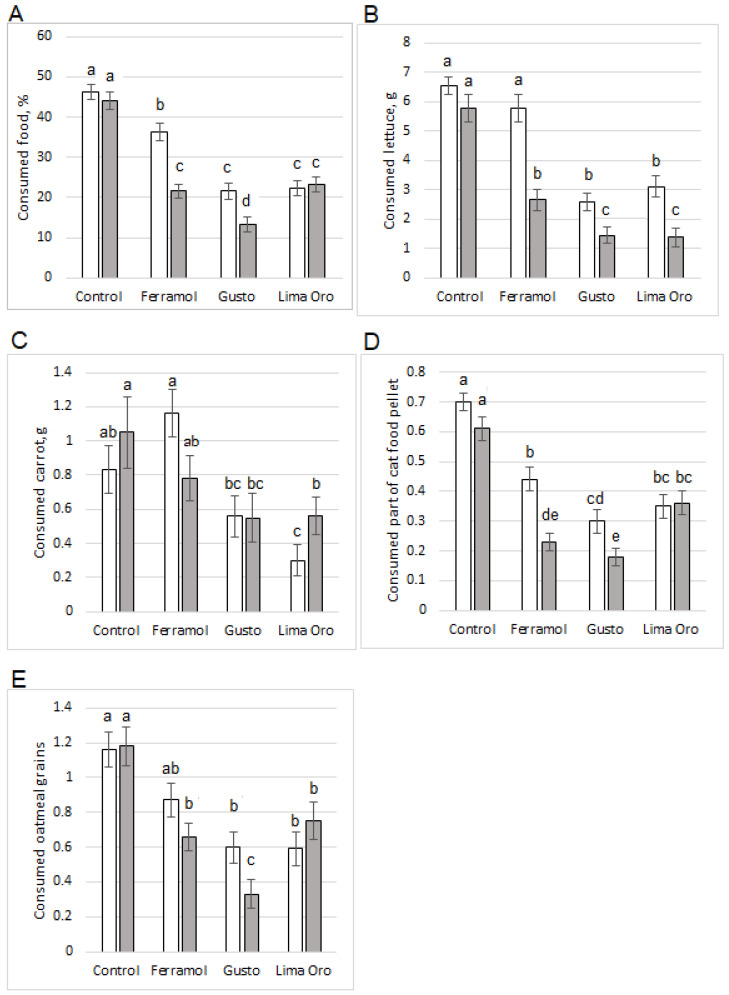
Mean (± standard error of the mean) food consumption by slugs over the seven-day experiment: (**A**) all food (mean total percentage); (**B**) lettuce (mean total g); (**C**) carrot (mean total g); (**D**) mean of total number (or part) of cat foot pellets; (**E**) mean of total number (or part) of oatmeal grains. For each bar, treatments with the same letter are not significantly different. Gray bars indicate treatments with one pellet, while black bars indicate treatments with five pellets. Means with different letters are significantly different (Tukey’s HSD, *p* < 0.05).

**Figure 4:**
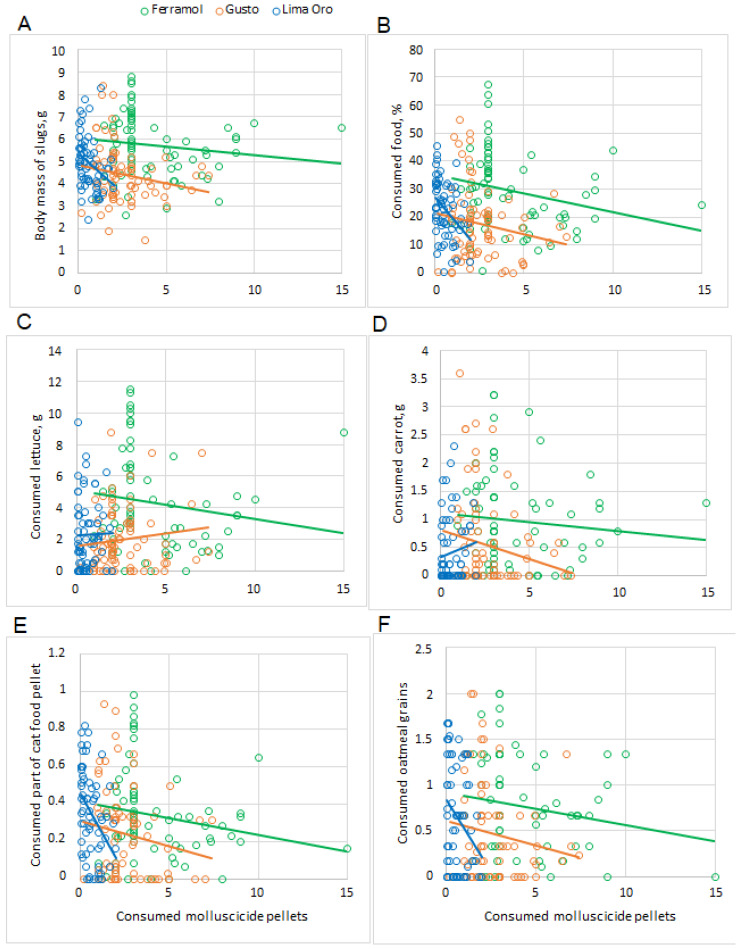
Body mass and food consumption of slugs after the 7-day treatment (for both designs) plotted with trend lines against the number of molluscicide pellets consumed: (**A**) body mass, g; (**B**) all food, %; (**C**) lettuce, g; (**D**) carrot, g; (**E**) number (or part) of cat food pellets; (**F**) number (or part) of oatmeal grains.

Notably, the decreased food consumption induced by the different molluscicides was only partially dependent on the number of pellets ingested (Figure 3 and Figure 4). For example, the slugs that ate seven Gusto pellets or two Lima Oro pellets had very high lettuce and oatmeal consumption (similar to slugs that ate fewer pellets). The consumption of four Gusto pellets, one Lima Oro pellet, or six Ferramol pellets reduced slug herbivory by half. Only one slug consumed all 15 pellets containing iron phosphate (Ferramol). Nevertheless, it ate a similar amount of food as the other slugs that consumed 1–9 pellets. Moreover, when examining different food kinds, lettuce consumption by this individual increased compared to other food types (Figure 4). There was a weak positive correlation between ingestion of the lowest numbers of Ferramol pellets (1–3) and lettuce consumption (Kendall tau-b correlation coefficient = 0.36, *p* < 0.001) (Table 4, Figure 4C). The mean percentage of total food consumption for slugs given five Ferramol pellets decreased by more than two-fold compared to slugs given one pellet (Figure 4B).

There was a weak positive correlation between the number of ingested Gusto and Lima Oro pellets (five pellet design) and lettuce consumption (Kendall tau-b correlation coefficient = 0.41; *p* < 0.001) (Table 4, Figure 4C). Notably, there was a mean decrease in lettuce consumption for both designs: slugs given one pellet consumed significantly less than those given five pellets (*t* = 2.01, *p* < 0.05 for both molluscicides) (Figure 3B).

With regard to the one-pellet design, the molluscicides with different amounts of metaldehyde did not significantly differ in decreasing total food consumption (*t* = 0.29, *p* = 0.991). However, Gusto (with 3% metaldehyde) was significantly more effective compared to Lima Oro (with 5% metaldehyde) in the five-pellet design (*t* = 3.67, *p* = 0.002) (Figure 3A). This effectiveness was obvious in the consumption of cat food (*t* = 4.01, *p* = 0.001) (Figure 3D) and oatmeal grains (*t* = 3.43, *p* = 0.005) (Figure 3E), but it was not in the consumption of lettuce (*t* = 0.375, *p* = 0.982) or carrots (*t* = 0.43, *p* = 0.972) (Figure 3B,C).

## 4. Discussion

We assessed the molluscicide activity of metaldehyde-baited (Gusto, 3% and Lima Oro, 5%) and iron-phosphate-baited (Ferramol, 1%) pellets sold in Lithuania. Data from laboratory treatments may not always be directly applicable to field conditions, but they do provide useful information on the potential of different baits.

Our results showed very low total mortality (2.1%, *n* = 240) in *A. vulgaris* for all of the molluscicides studied. The slugs that died were part of the metaldehyde treatment groups, so the mortality for that molluscicide was 3.1% (*n* = 160). Brooks et al. [26] reported that the mortality from 6% metaldehyde (0.09 g pellets for three slugs were used) was very low (14%) at the end of the seven-day experiment when slugs could select pellets and wheat seeds. Such differences in mortality may have been caused by the slug species (we studied *A. vulgaris*, Brooks et al. [26] examined *D. reticulatum*), food, or care differences (our containers were changed every two days, the slugs were irrigated, and food was replaced; Brooks et al. [26] left slugs for seven days without additional cleaning or irrigation). Kozłowski et al. [24] found that 1% iron phosphate rate of 1 g/m^2^ (0.07 g pellets for 2 slugs) led slugs to 20% mortality after seven days of treatment, while a rate of 2.5 g/m^2^ (0.17 g pellets for 2 slugs) led to 50% mortality (after 9–11 days of treatment). Comparatively, 5% metaldehyde provided at 0.4 g/m^2^ (0.03 g pellets for two slugs) led to 30% mortality after seven days of treatment. Note that those experiments used juvenile *A. vulgaris* (average weight 1.97 g), while our experiments used adult *A. vulgaris* (average weight 4.5 ± 0.69 g). Grubišić et al. [25] mentioned that treatments with 5% metaldehyde led to *A. lusitanicus* (or *A. vulgaris*) death between 9 and 24 days. Therefore, the duration of experiments should be extended in anticipation of the development of such research in the future. Furthermore, Chabert [27] reported that the effectiveness duration of metaldehyde pellets is about one week under moist conditions. The formulations and durability of pellets have changed much since that paper was published, but the review of the molluscicide metaldehyde [28] shows that the metaldehyde degradation in soil varies between 3.17–223 days depending on environmental conditions. Irrigation, which simulates the frequent rain, fog, and dew, had perhaps the greatest impact on slug survival. Literature data have shown that wet conditions can accelerate the degradation of molluscicide pellets and increase the recovery of slugs from poisoning [15,16,17,18,20,21,22].

Previous research has shown that *A. lusitanicus* (or *A. vulgaris*) can feed on 103 plant species [29]. Brassicas, lettuce, beetroot, carrots, beans, strawberries, numerous ornamental flower plants, and some weeds are among the most damaged [2]. These findings have led us to the idea that slugs, after tasting molluscicides and feeling unwell, can change their food source, such as switching from drier seed food to juicier leafy vegetables or root gnawing, or vice versa. Thus, we created conditions in which slugs could choose the number of pellets and different types of food that they consume: leafy plants (lettuce), root vegetables (carrot), a cereal-based diet (oatmeal), etc. Additionally, *A. vulgaris* willingly eat other dead slugs, so we offered animal food—cat food—as one of the food choices. Our results showed that total herbivory after the seven-day treatment with molluscicides was significantly decreased (*p* < 0.05) from 21.61% (Ferramol, one pellet design) to 53.29% (Gusto, one pellet design) and from 45.95% (Lima Oro, five pellets design) to 68.79% (Gusto, five pellets design). The ability to choose different kinds of food revealed that the choice of food after exposure to molluscicides was slightly different, although there was a significant decrease in food consumption in most cases (especially for animal food). Molluscicide consumption did not have a reliable effect (*p* > 0.05) on carrot consumption (except Lima Oro and Gusto, five pellets design). There were interesting data for lettuce consumption; immediately after metaldehyde molluscicide introduction, the consumption of lettuce decreased sharply, but there was a subsequent smoothing effect when the consumption of lettuce gradually increased again with an increasing dose of molluscicide (there was a weak positive Kendall’s tau-b correlation, Table 4). A smoothing effect has also been mentioned in some other studies [4,17], but it was usually explained by the effect of moisture or watering. Moisture was also high in our studies, so although we ruled out the influence of food choice on this occasion, further research is needed on this topic. For future studies, experiments could move from the laboratory to gardens and investigate the differences in damage to various plants after exposure to different molluscicides. In contrast to the results of other studies [30,31], we found a significantly smaller effect on lettuce consumption by an iron phosphate-based molluscicide (Ferramol) compared to metaldehyde-based molluscicides (Gusto, Lima Oro). These results are similar to those of Speiser and Kistler [32].

The recommended application rate usually is not calculated per slug; rather, it is determined per square meter or other unit of area, which may contain different numbers of slugs. Data in the literature have shown that the recommended rates of pellets was not efficient in some areas with high densities of *A. vulgaris* [17]. Metaldehyde pellets were not effective when the density of *D. reticulatum* exceeded 33 slugs per square meter [18].

Our results showed the low effectiveness of the most widely-used molluscicides compared to some other research [18,24,25,26], a phenomenon that may be due to several factors. One of the main reasons for differences could be different study design, as slugs in our study had the ability to choose the number of pellets next to the foods. Other studies likely used other feeding methods, such as injection [33]. Furthermore, in the majority of studies, active ingredients are provided without feeding the slugs at all [15]. One must also remember that bait pellets have different concentrations of active ingredients (metaldehyde or iron phosphate), as well as unknown attractants, coloring, and antifungal agents; their performance may vary and affect their effectiveness as molluscicides. Even pellet size can affect consumption; larger metaldehyde molluscicide pellets were less effective and less attractive for *Deroceras reticulatum* [18].

In general, the use of the lower application rate of molluscicides could raise resistance of mollusks towards tested chemicals. Salmijah et al. [34] found the development of resistance in snails *Achatina fulica* (Férussac, 1821) and *Bradybaena similaris* (Férussac, 1821) towards metaldehyde when half of the lethal concentration of baited metaldehyde (3% and 0.475%) was given repeatedly every day to *A. fulica* and *B. similaris*, respectively. The mortality rates of snails decreased significantly only after the fifth and seventh times, but this is debatable and requires more research because some other studies have indicated that molluscicides are more effective when they are used multiple times [25]. On the other hand, we have not received an expected mortality since the first days of treatment, so we cannot talk about the development of resistance.

During our seven-day study, no slug (*n* = 240) reached the recommended rate and did not consume 16 pellets (or 2.5 g/m^2^) of Ferramol, 10 pellets (or 0.6 g/m^2^) of Gusto, or four pellets (or 0.7 g/m^2^) of Lima Oro (Figure 4). The highest number of pellets reached during this study per slug were: 15 pellets (or 2.34 g/m^2^) of Ferramol, seven pellets (or 0.42 g/m^2^) of Gusto, and two pellets (or 0.35 g/m^2^) of Lima Oro. The median consumption of molluscicide pellets during the first day of exposure (Figure 2) showed that the majority of slugs ate slightly more than half of the single dose per square meter (Ferramol). Our results showed that the most “unpalatable” pellets contained 5% metaldehyde (Lima Oro); when given the ability to eat five pellets at once during a seven-day treatment, most slugs tasted only one. Furthermore, 25% of the slugs did not taste any Lima Oro pellets. Other recent research also showed that the highest metaldehyde concentration (5%) produced the highest proportion of un-poisoned slugs, suggesting the highest level of pellet rejection [21]. Gusto pellets, with 3% metaldehyde, were more acceptable (some slugs consumed three pellets) and more effectively protected the plants (Figure 4). Thus, reducing this product application rate in half would not reduce efficiency, but it would significantly help to protect the environment.

Until sufficient effective nontoxic solutions are identified [14], our results suggest that new applications of molluscicides could be useful; slugs should be monitored and the application rate should be adjusted according to the number of slugs and to the established tendency of slugs to eat a certain amount of specific molluscicide pellets. Especially since we get a significant effect of time, this becomes even more important as it shows that in the second half of the summer, before the laying of eggs, the roughness of the slugs naturally decreases, and, therefore, the amount of poison eaten is reliably lower. Considering the same metaldehyde-baited (Gusto, 3% and Lima Oro, 5%) and iron-phosphate-baited (Ferramol, 1%) pellets, the doses required to reduce herbivory by half are: four pellets (or 0.24 g for Gusto), one pellet (or 0.18 g for Lima Oro), and six pellets (or 1 g for Ferramol) per slug. According to the published data, the density of *A. vulgaris* slugs in Lithuania varies from 1 to 23 individuals per square meter [1]. It is not possible to use a larger dose of molluscicide pellets than written in product application instructions in order to prevent environment contamination by molluscicides. However, in areas with a low density of slugs, smaller application rates could be applied according to our calculations, which could reduce the number of uneaten pellets.

## 5. Conclusions

This study confirmed that the chemicals are still a good option in reducing the damage to plants caused by slugs, but their efficacy was not as it should be; this effect was quite low compared to information provided by other researchers. During the seven-day treatments in wet conditions (typical for temperate climate zone), adult *A. vulgaris*, who were allowed to freely choose among the available molluscicide pellets and food kind, reduced damage to plants in half but did not stop eating, and the mortality rate was negligible (2.1%). As our finding about the role of molluscicides contradicts other findings [24,25], further studies on the role of freely choosing the amount of molluscicides and plant diversity on slug abundance would help.

Whilst slugs are mostly controlled using chemical molluscicide products, their detrimental environmental effects can be reduced by application rate reduction. We recommend doing this in two ways: (1) to use molluscicides with a lower metaldehyde percentage in pellets, as Gusto (3%) was more attractive and more edible than Lima Oro (5%) with the same effect of food consumption diminishing; (2) to monitor the slugs and use the application rate counted according to the number of slugs and to the ability of slugs to consume a certain amount of molluscicide pellets. Considering the same metaldehyde-baited (Gusto, 3% and Lima Oro, 5%) and iron-phosphate-baited (Ferramol, 1%) pellets, the number of pellets required to reduce herbivory by half are: four pellets (or 0.24 g for Gusto), one pellet (or 0.18 g for Lima Oro), and six pellets (or 1 g for Ferramol) per slug.

## Figures and Tables

**Table 1:** Slug control measures used in treatments.

Product	Active Ingredient, Concentration	Recommended Rate, g/m^2^	A single Dose, 1 or 5 Pellets, g
Ferramol^®^ (Neudorff, Germany)	Iron phosphate, 1%	2.5 (16 pellets)	0.16 or 0.8
Gusto^®^ (Adama, The Netherlands)	Metaldehyde, 3%	0.6 (10 pellets)	0.06 or 0.3
Lima Oro^®^ (Sharda, Spain)	Metaldehyde, 5%	0.7 (4 pellets)	0.18 or 0.9

**Table 2:** Timetable of activities and measurements for the experiment during one treatment.

	Day	1	2	3	4	5	6	7
Activity								
Trial setup		x						
Addition of new pellet(s)		x		x		x		
Addition of new food portion		x		x		x		
Cleaning and moistening the slug		x		x		x		
Assessment of slug herbivory (separately for each food type)				x		x		x
Assessment of pellet ingestion by the slug				x		x		x
Toxicity assessment				x		x		x
Weighing		x		x		x		x
Removal of old pellets				x		x		x
Removal of old food portion				x		x		x
Cleaning container				x		x		x
Slug removal								x

**Table 3:** Slug herbivory and slug weight in response to slug control (SC), time (T), and design (D) or their interactions (Int.). Df = degrees of freedom, Sum Sq = sum of squares, Pr (>F) = *p* value. Asterisks indicate significant effects: * *p* < 0.05; ** *p* < 0.01; *** *p* < 0.001.

Factors	Df	Sum Sq	F Value	Pr (>F)	Sum Sq	F Value	Pr (>F)
Herbivory					Weight		
SC	3	3.027	87.999	<0.001 ***	242.07	61.083	<0.001 ***
D	1	0.318	27.754	<0.001 ***	39.31	29.757	<0.001 ***
T	1	0.478	41.691	<0.001 ***	40.83	30.909	<0.001 ***
SC-D Int.	3	0.243	7.077	<0.001 ***	21.28	5.370	0.001 **
SC-T Int.	3	0.107	3.110	0.0269 *	5.35	1.351	0.258
D-T Int.	1	0.015	1.284	0.258	11.51	8.711	0.003 **
SC-D-T Int.	3	0.073	2.129	0.0969	7.69	1.941	0.123

**Table 4:** Kendall rank correlation coefficients between slug mass, food consumption, and percentage of damaged slugs and the number of bait pellets consumed. The number next to the molluscicide name indicates the number of pellets (one or five) given every two days (a total of three times). Asterisks indicate significant effects: *p* < 0.05.

Control Measure	Slug Mass	Damaged Slugs	Lettuce	Carrot	Cat Food	Oat-Meal	All Food
Ferramol 1	+0.34 *	−0.18	+0.36 *	−0.04	+0.23	+0.21	+0.39 *
Gusto 1	−0.14	−0.09	+0.15	−0.09	−0.14	−0.22	−0.16
Lima Oro 1	−0.49 *	+0.54 *	−0.24	−0.11	−0.42 *	−0.21	−0.37 *
Ferramol 5	+0.21	+0.16	+0.21	+0.12	+0.16	−0.06	+0.11
Gusto 5	+0.06	+0.42 *	+0.16	−0.12	−0.02	+0.14	+0.06
Lima Oro 5	−0.08	+0.41 *	+0.41 *	+0.21	−0.21	−0.31 *	−0.15

## Data Availability

The data presented in this study are available on request from the corresponding author.
